# Physical Education and Blood Lipid Concentrations in Children: The LOOK Randomized Cluster Trial

**DOI:** 10.1371/journal.pone.0076124

**Published:** 2013-10-25

**Authors:** Richard D. Telford, Ross B. Cunningham, Paul Waring, Rohan M. Telford, Lisa S. Olive, Walter P. Abhayaratna

**Affiliations:** 1 National Institute of Sports Studies, Faculty of Health, University of Canberra, Canberra, Australian Capital Territory, Australia; 2 Clinical Trials Unit, Academic Unit of Internal Medicine, Canberra Hospital, Garran, Australian Capital Territory, Australia; 3 Fenner School for Environment and Society, Australian National University, Canberra, Australian Capital Territory, Australia; 4 ANU College, Australian National University, Canberra, Australian Capital Territory, Australia; 5 Centre for Research and Action in Public Health, Faculty of Health, University of Canberra, Bruce, Australian Capital Territory, Australia; 6 Research School of Psychology, Australian National University, Canberra, Australian Capital Territory, Australia; 7 Medical School, College of Medicine, Biology and Environment, Australian National University, Canberra, Australian Capital Territory, Australia; Universidad Europea de Madrid, Spain

## Abstract

**Background and Objectives:**

Elevated blood lipids during childhood are predictive of dyslipidemia in adults. Although obese and inactive children have elevated values, any potentially protective role of elementary school physical education is unknown. Our objective was to determine the effect of a modern elementary school physical education (PE) program on the blood lipid concentrations in community-based children.

**Methods:**

In this cluster-randomized controlled trial, 708 healthy children (8.1±0.3 years, 367 boys) in 29 schools were allocated to either a 4-year intervention program of specialist-taught PE (13 schools) or to a control group of the currently practiced PE conducted by generalist classroom teachers. Fasting blood lipids were measured at ages 8, 10, and 12 years and intervention and control class activities were recorded.

**Results:**

Intervention classes included more fitness work and more moderate and vigorous physical activity than control classes (both p<0.001). With no group differences at baseline, the percentage of 12 year-old boys and girls with elevated low density lipoprotein cholesterol (LDL-C, >3.36mmol.L^−1^,130 mg/dL) was lower in the intervention than control group (14% vs. 23%, p = 0.02). There was also an intervention effect on mean LDL-C across all boys (reduction of 9.6% for intervention v 2.8% control, p = 0.02), but not girls (p = 0.2). The intervention effect on total cholesterol mirrored LDL-C, but there were no detectable 4-year intervention effects on high-density lipoprotein cholesterol or triglycerides.

**Conclusions:**

The PE program delivered by specialist teachers over four years in elementary school reduced the incidence of elevated LDL-C in boys and girls, and provides a means by which early preventative practices can be offered to all children.

**Trial Registration:**

Australia New Zealand Clinical Trial Registry ANZRN12612000027819 https://www.anzctr.org.au/Trial/Registration/TrialReview.aspx?id=347799.

## Introduction

There is compelling evidence that elevated lipid concentrations during childhood are predictive of dyslipidemia in adults [1], [2] and are associated with an increased risk of cardiovascular disease in later life [3], [4], [5]. Given that overweight [6] and insufficiently active children [7] are more likely to possess elevated lipid levels, the challenge is to identify and implement strategies capable of reducing obesity and increasing physical activity across the general community.

An obvious opportunity to mount such a challenge in children is during their attendance at school, especially as physical education (PE) is usually a mandatory component of the elementary (primary) school curriculum. However, whilst modern accurate data were not available from Australian jurisdictions, few specialist PE teachers appear to be employed in the public elementary school systems. For example, in 30 randomly selected primary schools in the local jurisdiction, where planning and educational philosophy were similar to most other states, there were 2 specialists teaching PE. Consequently, the responsibility for delivering a PE program falls on the general classroom teachers, but along with increasing pressure to improve academic standards, generalist teachers themselves have reported difficulties in teaching PE effectively [8].

The primary objective of this investigation was to determine whether a well-constructed program of specialist taught PE over the final four years of elementary school could benefit lipid profiles. A secondary objective was to determine whether any such effect could be explained by changes in physical activity, fitness or body composition. This was part of a larger multidisciplinary study, the Lifestyle of our Kids (LOOK) study [9], areas of investigation including cardiovascular risk [10], body composition [11], academic performance[12], bone geometry [13], body image [14], insulin resistance [15], and pediatric blood reference ranges [16]. Because a control group devoid of PE was not possible, our control group consisted of children whose PE was delivered by general classroom teachers; and to highlight differences in the two programs we employed a systematic method of class observation to classify key characteristics. Consequently we were able to test the null hypothesis that there is no difference in the effectiveness of the specialist PE program and the current practice of classroom teacher PE to modify lipid profiles.

## Methods

This study in its entirety was approved by the Ethics Committee for Human Research at the Australian Institute of Sport; parental consent was obtained for all measures and children understood their participation was entirely voluntary.

### Participants and Design

**Figure 1:**
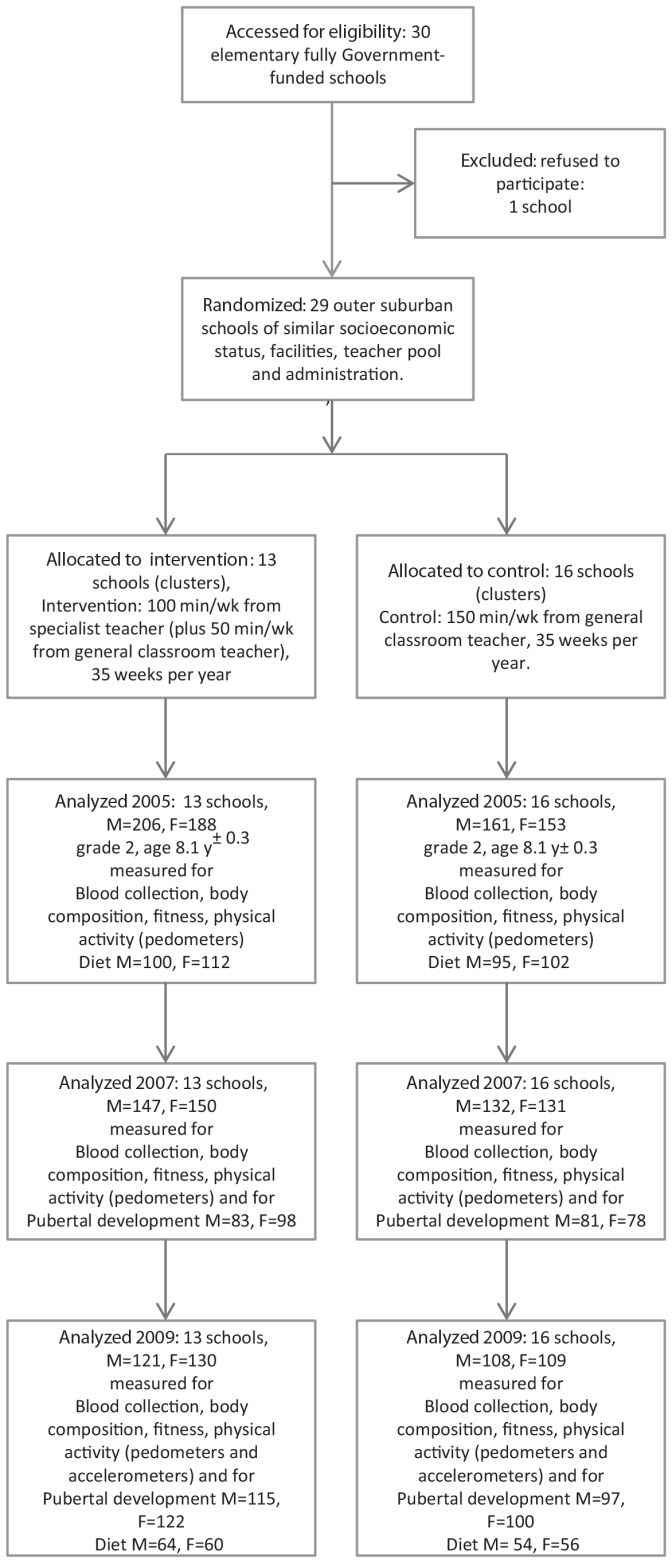
Flow chart of study, showing numbers of observations in the intervention and control groups. Details of the attrition are summarized in the text.

The general schema of this cluster-randomized trial is outlined in Figure 1. The LOOK study began in February 2005; the measurements for the current section of work took place during September through November in 2005, 2007 and 2009.

Grade 2 children attending fully government-funded elementary schools in Australian suburbs of mid-range socio-economic status (details below) were recruited through invitation to principals; 29 schools were required to obtain the maximum number of 850 participants measurable within a school term of 10 weeks. Class sizes were typically around 25 children and all classes consisted of approximately equal number of boys and girls. As an introductory summary of our design, the intervention consisted of two 50 minute classes of PE conducted by 3 visiting specialist PE teachers, assigned to 13 schools. This replaced 100 minutes of the current curriculum requirement of 150 min/week PE, a requirement all teachers reported to have satisfied. The control group consisted of the usual practice of PE conducted by generalist classroom teachers. To avoid any potential contamination of methodology, intervention and control groups were allocated into separate schools, hence our cluster-randomized design. A condition of inclusion was for parents to indicate in writing that children were in good health, able to participate in vigorous physical activity, willing to provide a venous blood sample, undertake a DEXA scan, participate in fitness and physical activity assessments and likely to remain in school for the four years duration of the study. Notice of acceptance and written informed consent was received from the parents of 708 eligible children, 73% of those invited. Following acceptance, two members of the research team used the sealed envelope system to allocate schools to the intervention and control groups. A CONSORT checklist is attached as Checklist S1.

### The intervention

The PE intervention was provided by the not-for-profit Bluearth Foundation. Their staff were university trained qualified PE teachers with further specialized training from postgraduate trained PE and psychology consultants to the Foundation. Its underlying philosophy and programs are outlined through its website [17], and summarized in Table 1. This program, with a modus operandi of physical activity without nutritional or health instruction, has been in operation in several Australian states for approximately 10 years, its content meeting the respective jurisdiction curricula requirements. The beneficial effects of two years of this program on body composition and academic results have already been reported [12]; the current investigation addresses its effect on blood lipids. A comparison of the intervention and control PE classes was made using a validated system of observing fitness instruction time (SOFIT) [18] which involves a group of trained observers recording the duration of particular categories of physical activity of randomly selected children, as well as the teacher's behavior.

**Table 1:** Characteristics of the Physical Education intervention^a^.

Provider	The Bluearth Foundation, a not-for-profit company.
**Program objectives**	To create an all-inclusive, enjoyable, challenging yet non-threatening environment of physical activity.
**Child-related Objectives**	Personal, social and physical development through movement challenges.
**Teacher-related Objectives**	Ongoing in-class professional development to provide generalist classroom teachers with practical PE teaching skills and resources.
**Activity Elements**	
**Coordination and Agility**	Challenges include crawling, climbing, running, hopping, stepping, skipping, rope-skipping and jumping.
**Skill Activities**	Individual and group practices to develop motor skills of hitting, kicking, throwing, juggling and catching with a variety of objects.
**Dynamic Movement**	Gymnastic activities to develop rhythm and balance. For example, double leg jump squat to single leg landing.
**Games**	Designed to promote aerobic fitness, cooperation and teamwork, problem solving and healthy attitudes to competition.
**Core Movement**	Include yoga-like practices to develop muscular strength, flexibility, balance and postural control, and quiet times for reflection.

a a detailed description of the program and its components can be found at www.bluearth.org.

### Anthropometry, cardiorespiratory fitness and physical activity

Body composition (lean and fat mass) was assessed by dual energy x-ray absorptiometry at (DEXA; Hologic Discovery QDR, Bedford, MA, USA) in the hospital venue. Total body scans were analyzed using Hologic QDR System Software Version 12.4 to calculate total mass, fat mass and percent body fat. Body mass was also measured using portable electronic scales to 0.05 kg and height by a portable stadiometer to 0.001 m. The 20 m shuttle run was conducted at the school to estimate cardiorespiratory fitness from the number of successfully completed 20 m stages at progressively faster speeds, this being well-established as a field test [19]. Children wore pedometers (Walk 4 Life; Plainfield, IL USA) on their hip for 7 consecutive days and a physical activity index was derived. This index was the square root of the average daily steps per day, transformed to meet the linearity requirements of our statistical model, and adjusted for missing data and days of the week that did not meet inclusion requirements as previously described [20]. Physical activity was also assessed in grades 5 and 6 using accelerometers during the school days (Actigraph GTIM, Pensacola, FL, USA), these not being available to the study in the first two years. Moderate physical activity was defined at 2296 to 4012 counts/min and vigorous physical activity at greater than 4012 counts/min, based on recommendations [21] using an epoch of 60 seconds, and data were analyzed using Meterplus software (San Diego State University and Actigraph, Pensacola, FL, USA). The first day's data did not represent full days and were discarded for both accelerometer and pedometers.

### Blood chemistry

Venous blood was drawn from the forearm of overnight fasted children (water only) between 8.00am and 9.30am at the schools during the measurement period of grades 2, 4, and 6 (2005, 2007 and 2009 respectively) as indicated in Figure 1. Total cholesterol and triglyceride concentrations were measured at the Canberra Hospital Pathology laboratories using the Architect Ci8200 (Abbott laboratories, IL 60064 USA). LDL-cholesterol was calculated by the Friedwald formula [22]. All methods were carried out following the manufacturers' instructions and performed within acceptable limits as determined by internal quality control.

### Dietary Intake

In grade 3, total energy and macronutrient intakes were examined using a one-day dietary record based on a previously described validated method [23] where children, parents and teachers combined to record intake over a 24-hour period, with a follow-up interview to check details. In grade 6, the methodology was essentially the same, but based on the 2007 Australian National Children's Nutrition and Physical Activity Survey [24] and included two 24 hr recalls, (one school day and one non-school day). Analysis of the data was carried out using the FoodWorks Professional ™ software system (version 2007, Xyris, Brisbane Queensland).

### Medical History, Pubertal and Socioeconomic Status

A history of heart disease, stroke or elevated blood cholesterol in parents or grandparents was determined by questionnaire to the parents. Pubertal development was a self-assessment of “Tanner” stage [25] (pubic hair, and genital development for males, breast development and date of menarche for females) as previously described [26]. In grade 4 this occurred at home with parental guidance, and in grade 6 the venue was a private room in a hospital setting with guidance from an experienced teacher. The socioeconomic status of each school suburb was accessed through Australian government Bureau of Statistics [27]. We used a published socioeconomic index that designated advantage (high values) and disadvantage (low values) derived from variables such as income, educational attainment, and employment. The mean and standard deviation of this index for the suburbs in our study (1085±40 and range 982–1160) was higher (with a smaller range) than Australia-wide (980±84, 598–1251).

All the technicians, with the sole exception of one involved with physical activity and fitness measurements, were blinded as to which children were in the intervention and control groups.

### Statistical Methods

Statistical modeling was used to determine whether the specialist-taught PE (intervention) had any effect on lipid levels in comparison with the usual PE (control) program delivered by classroom teachers. The model needed to account for the complexity of factors that influenced lipid concentrations at both the child and school (cluster) levels. Potential explanatory variables explored for inclusion in the model were sex, school grade (or age), weight, percent body fat, physical activity, energy intake and macronutrient intake, pubertal development, medical history and socioeconomic status. The model fits within the general framework of general linear mixed models. Statistical significance of an effect was assessed by calculating adjusted Wald statistics [28]. Where necessary, variables were scaled by square roots or natural logarithms to better meet linearity assumptions. General model checking procedures were routinely used to identify aberrant data and to check the model assumptions. In addition to the above model, differences between mean logit percentage values of SOFIT class observational data and of elevated lipid concentrations were determined with a formal linear mixed model analysis adjusting for random school effects. A further application of our model was to determine the effect of the intervention on the incidence of elevated (at risk) LDL-C. Data were converted to a binary indicator response (1 for elevated LDL-C or 0 normal). Linear logistic regression assessed the significance of difference in the change of the probabilities in the two groups. All procedures were carried out using the statistical package Genstat version13 (VSN International Ltd, Oxford, UK), which accounts for any imbalance if a child was not measured in all years, listwise deletion applying if a covariate value was missing.

## Results

### Baseline characteristics

Table 2 shows the characteristics of the participants at baseline, and there were no significant differences between the intervention and control groups in any of the measurements. Table 3 presents unadjusted values classified by elementary school grade and gender.

**Table 2:** Comparison of the intervention and control groups at baseline.

	Males 2005, grade 2				Females 2005, grade 2			
	Intervention		Control		Intervention		Control	
	n = 206		n = 161		n = 188		n = 153	
	Mean	SE	Mean	SE	Mean	SE	Mean	SE
**Age, y** a	8.1	0.3	8.1	0.3	8.1	0.3	8.1	0.3
**Ht, cm**	130.5	0.4	129.9	0.4	128.4	0.4	129.1	0.4
**Wt, kg**	29.8	0.4	29.0	0.4	28.6	0.4	28.8	0.4
**BMI, kg/m^2^**	16.9	0.2	17.0	0.2	17.2	0.2	17.2	0.2
**SqrtPA, steps/d**	108.5	0.9	107.5)	0.9	96.2	0.9	98.5	0.9
**SqrtCRF, stages**	2.0	0.03	2.0	0.03	1.8	0.03	1.9	0.03
**%Body Fat**	22.8	0.5	22.5	0.5	27.9	0.5	28.0	0.5
**LnEnergy, kJ/d** b	8.9	0.2	8.8	0.2	8.9	0.3	8.7	0.2
**LnFat, kJ/d** b	7.8	0.1	7.7	0.1	7.8	0.1	7.7	0.1
**LnCHO, kJ/d** b	8.3	0.4	8.2	0.4	8.3	0.4	8.2	0.3
**LnSugar, kJ/d** b	7.5	0.1	7.4	0.1	7.5	0.1	7.4	0.1

The abbreviation PA is physical activity, CRF is cardiorespiratory fitness, Energy is energy intake, Fat is fat intake, CHO is carbohydrate intake, Sugar is sugar intake; these were scaled by square root or the natural logarithm to meet linearity assumptions. There were no significant differences between intervention and control groups for any variable in either sex (P>0.3 for all).

a the age for each child varied across the measurement period of 10 weeks.

b 164 (83 intervention) boys and 176 (98 intervention) girls completed the dietary records.

**Table 3:** Unadjusted values of body size, percent body fat, physical activity, fitness and pubertal development, medians and percentiles classified by elementary school grade and gender.

		Grade 2M = 367 F = 341			Grade 4M = 279 F = 281			Grade 6M = 229 F = 239		
		5%	Med	95%	5%	Med	95%	5%	Med	95%
**Ht**	F	120.1	**128.6**	137.3	130.3	**140.5**	150.3	141.5	**154.1**	164.7
**cm**	M	120.6	**130.2**	139.4	130.9	**141.9**	152.0	141.2	**153.5**	166.3
**Wt**	F	21.7	**27.3**	39.8	26.4	**34.5**	53.0	32.7	**45.00**	66.2
**kg**	M	22.5	**28.0**	38.6	27.2	**35.5**	50.3	33.2	**44.75**	66.5
**BMI**	F	14.1	**16.6**	22.4	14.5	**17.7**	24.7	15.2	**19.0**	26.2
**kg/m^2^**	M	14.2	**16.5**	21.1	14.7	**17.6**	23.7	15.5	**18.9**	25.3
**PA** a	F	80.1	**97.3**	113.4	78.8	**93.5**	110.7	77.1	**91.4**	106.5
**sqrtsteps/d**	M	90.4	**107.9**	126.9	84.5	**102.2**	120.1	78.5	**98.0**	116.5
**CRF** b	F	2.2	**3.2**	5.4	2.6	**4.1**	7.3	3.0	**5.2**	8.9
**run stage**	M	2.2	**4.1**	6.9	2.8	**5.4**	8.7	2.9	**6.2**	10.1
**%BF** c	F	19.2	**27.1**	39.5	18.5	**28.5**	41.9	18.3	**26.6**	39.5
	M	15.3	**21.8**	34.3	15.6	**23.9**	36.7	14.2	**23.5**	38.6
**Tanner** d	F	not assessed			1.0	**1.5**	2.6	1.5	**2.5**	4.0
**stage**	M				1.0	**1.5**	2.5	1.0	**2.5**	4.0

a PA is physical activity, the average daily steps per day;

b CRF is cardiorespiratory fitness, the number of stages reached in the multistage run;

c %BF is the percentage of body fat as determined by DEXA scan;

d Tanner stage is self-assessed pubertal stage ranking (N = 176 females, 164 males in 2007, 222 females and 212 males in 2009).

### Attrition

The numbers of observations are shown in Figure 1. The only children involved in this study were those who began in grade 2; the reduction from grade 2 to 4 was 148 children (21%) and from grade 4 to 6 it was 92 (16.5%). Approximately 70% of those who left the study left the project schools, 8% withdrew and technical difficulties or absences on test day accounted for remaining reduction in observations. A comparison of the baseline data from children who remained in the study with those who left revealed no evidence of any difference in the means of blood lipids, body weight, percent body fat or physical activity (all p>0.3, data not shown here). We have no reason to suspect attrition resulted in any study bias.

### Intervention and control programs

There were SOFIT observations of 194 PE lessons (97 intervention lessons). Average actual (face to face) teaching time per lesson was greater in the intervention group (44.1 vs. 33.2 min, respectively; p<0.001); as was the accumulated duration of moderate and vigorous physical activity (16.9 vs. 10.2 min; p<0.001); specific fitness-related activities (7.5 vs. 0.7 min; p<0.001); and games (11.2 v 3.1 min, p<0.001), with little difference in time spent on motor skills (4.1 v 3.0 min, p>0.2). The nature of activities in each group also differed. Physical activity and fitness work in the intervention group occurred incidentally, mainly through balance and coordination challenges and minor games whereas fitness activity in the control program occurred mainly through walk/runs and more traditional strength and flexibility exercises. Finally, intervention teachers participated more in class activities (11.7 v 2.0 min, p<0.001).

### Risk Categorization

We employed the American Heart Association (AHA) and American Association of Pediatrics (AAP) LDL-C cutoff point of 3.36 mmolL^−1^ (130 mgdL^−1^) [5], [29]. There were no significant gender differences (all p>0.1) in the incidence of any of the blood lipids. For LDL-C 19.7% of the boys and 16.3% of the girls possessed elevated values at age 12 years. However high-density lipoprotein cholesterol (HDL-C) data were more encouraging with only 1.7% and 2.8% of 12 year-old boys and girls respectively below the cutoff point of 0.9 mmolL^−1^ (35 mgdL^−1^), and percentages of elevated triglyceride concentrations (>4.52 mmolL^−1^, 400 mgdL^−1^) were low, at 1.3% and 0.5%. The percentages of children with elevated total cholesterol (>5.17 mmolL^−1^, 200 mgdL^−1^), at 16.5% for boys and 12.5% for girls respectively, reflected those for LDL-C.

### Intervention effect on blood lipids

**Figure 2:**
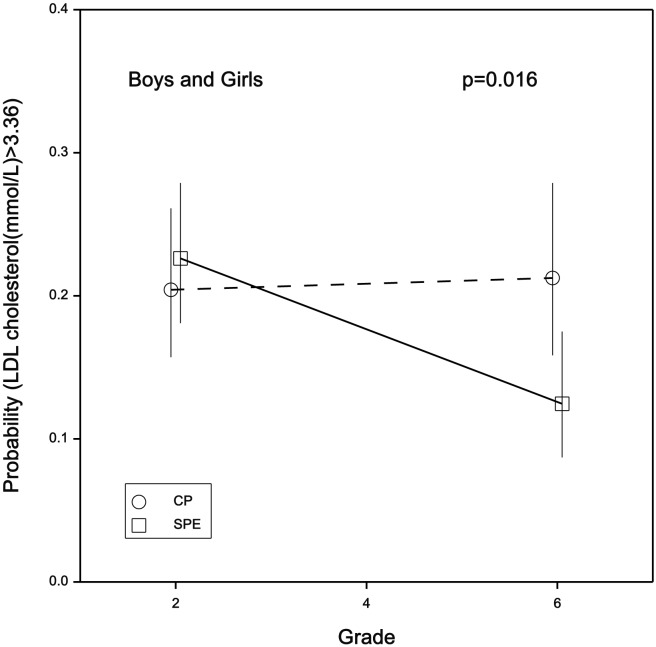
The probability of elevated (at risk) LDL-C at baseline and grade 6 in the intervention (SPE) and control (CP) groups, with least significant difference (lsd) and p-value for the intervention effect. The lsd provides a close approximation of the difference in means that is significant at p = 0.05.

With no gender interaction (p = 0.3) there was an intervention-induced reduction in the percentage of both boys and girls with elevated LDL-C. With no significant difference at baseline (24% and 22% for intervention and control groups respectively, p = 0.2), by grade 6 (age 12) there was a significantly lower incidence of elevated LDL-C in the intervention group compared with the control group (14% vs. 23%, p = 0.02). Figure 2 illustrates this effect in terms of the probability of a boy or girl having an elevated LDL-C, the intervention significantly reducing this probability (p = 0.016).

**Figure 3:**
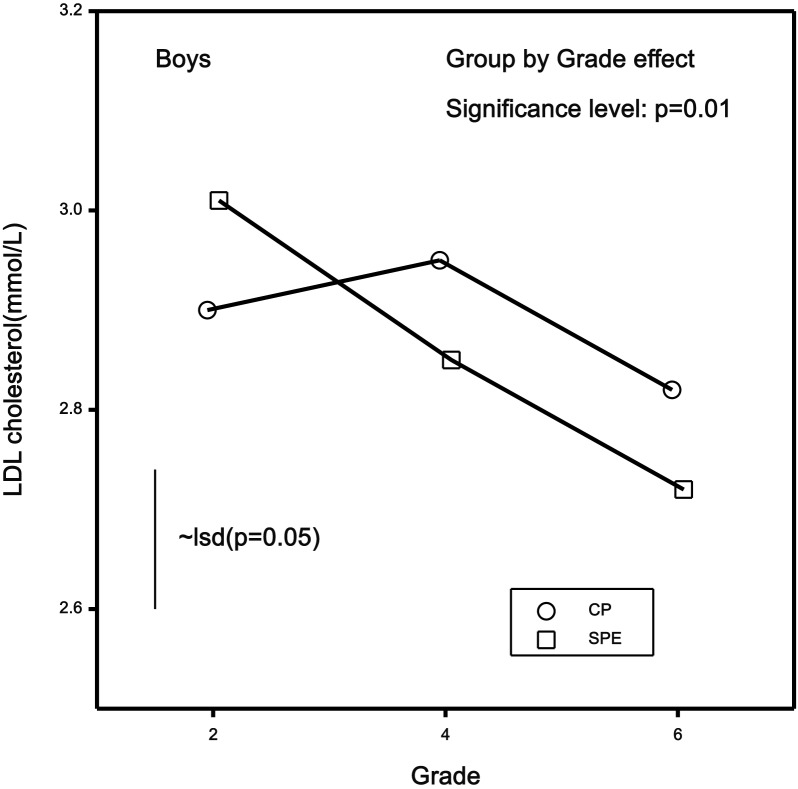
Mean LDL-C of the boys through grades 2, 4, and 6 for intervention (SPE) and control (CP) groups, with the significance of the intervention (group by grade) effect and least significant difference (lsd) for the means. The lsd provides a close approximation of the difference in means that is significant at p = 0.05.

There was also an intervention effect across mean LDL-C over the 4 years (elevated or otherwise) in the boys (p = 0.01) but not girls (p = 0.2). As shown in Figure 3, the intervention lowered mean LDL-C in the boys by 9.6% compared with 2.8% in the control group. The beneficial effects on total cholesterol mirrored the effects on LDL-C, but there was no evidence of an intervention effect on either HDL-C or triglycerides (p>0.3 for both). Table 4 presents the means and standard errors of each blood lipid, classified by intervention and control group, across all elementary school grades, with the probabilities of an intervention effect.

**Table 4:** Means and standard errors (SE) of blood lipid concentrations, classified by the Intervention (Int) and Control (Con) groups and elementary school grade, with probabilities (p) of the intervention effect.

				Males							Females				
		Grade 2		Grade 4		Grade 6			Grade 2		Grade 4		Grade 6		
		N = 206, 161		N = 147, 132		N = 121, 108			N = 188, 153		N = 150,131		N = 130, 109		
		Mean	SE	Mean	SE	Mean	SE	P	Mean	SE	Mean	SE	Mean	SE	P
**LDL-C, mmol/L**	**Int**	3.01	.07	2.85	.07	2.72	.09	.01	2.81	.07	3.25	.0	3.13	.10	.15
	**Con**	2.90	.07	2.94	.09	2.82	.10		2.76	.07	3.11	.09	3.12	.10	
**HDL-C, mmol/L**	**Int**	1.44	.04	1.46	.04	1.43	.05	.5	1.33	.03	1.34	.03	1.45	.05	.8
	**Con**	1.43	.04	1.45	.04	1.58	.05		1.30	.03	1.35	.03	1.48	.04	
**Log TG, mmol/L**	**Int**	−0.42	.04	−0.42	.05	−0.40	.07	.16	−0.27		−0.30		.004		.53
	**Con**	−0.43	.04	−0.50	.05	−0.33	.07		−0.28		−0.37		−0.07		

### Other Intervention Effects

Accelerometry during the school week days showed that for the boys but not girls, mean daily moderate physical activity was 17% greater in the intervention group (41.7 v 35.7 min/day, p = 0.02), with higher mean vigorous physical activity (9.7 v 8.4 min/day) but this was not significant (p = 0.3). On the other hand, the pedometer step counts records revealed no significant intervention effects (p>0.3 for both boys and girls). Furthermore, there was no evidence of an intervention effect on the running cardiorespiratory fitness test (p>0.3), and little evidence of any 4-year intervention effect on percent body fat (p = 0.2) or on the total daily energy or macronutrient intake (all p>0.3).

Of 543 families (270 girls, 273 boys) responding to the question as to whether any parent or grandparent of the child had a history of elevated cholesterol, heart disease or stroke; 62% of the girls and 63% of the boys responded positively, but inclusion in our model did not affect any of the intervention affects. Pubertal status was estimated by an average score for ratings of pubic hair rating and genital development (boys) and breast development (girls). The medians (5^th^–95^th^ percentiles) for intervention and control groups in grade 6 were identical; boys 2.5 (1.0–4.0), girls 2.5 (1.5–4.0). Adjustment for pubertal status did not change the level of any intervention effects on the blood lipids; nor did socioeconomic status or total energy, fat, carbohydrate or sugar intake.

## Discussion

The most important finding from this study was that two 50 minute classes/week of PE delivered by specialist teachers over 4 years of elementary school reduced the prevalence of elevated LDL-C in both boys and girls, and also reduced mean LDL-C of all the boys (but not girls). As these effects were measured relative to a control group of usual practice PE conducted by general classroom teachers, our data indicate that the specialist conducted PE offered a means of offsetting early signs of cardiovascular disease that was not available to most children undertaking PE in government schools in this jurisdiction.

A recent review [7] identified three intervention studies in teenagers [30], [31], [32] which produced little effect on any of the blood lipids, but the reviewers, after considering evidence from observational studies, suggested that 40 minutes of moderate physical activity for 5 days per week over a minimum duration of 4 months may be required to achieve effects on HDL-C and triglyceride in children, and that changes in fitness seemed to be required to achieve any effect. Our data suggest otherwise when the intervention is in the form of the specialized PE provided for this investigation. Only two 50 minute specialized PE classes per week were required to lower the prevalence of elevated LDL-C in boys and girls and to lower mean LDL-C and total cholesterol in all boys when undertaken over 4 years; and this took place without detecting any change in our running-based cardiorespiratory fitness test. Interestingly, in relation to the latter point, the converse has also been reported; improvements in fitness can occur without any effect on lipid levels [33].

As to why these specific effects were detected when no effects on lipids were found in other interventions [30], [31], [32], we point to four design characteristics of our study which may have contributed. Firstly, in comparison with the cited studies, the current investigation involved considerably more participants (468 complete results verses 12 to 38) and was therefore in a better position to detect effects. Secondly, our intervention involved specialized PE rather than a physical activity or training program. Thirdly, our intervention was conducted over 4 years rather than the shorter term (1 to 5 months) physical activity or training programs of the cited studies. Finally, our participants were in their pre-teenage mainly pre-pubescent years, and being generally younger than the teenagers involved in the cited studies, may have responded to the intervention differently.

As this specialist-taught PE program focused entirely on physical activity, and avoided any discussion of the children's body weight or their diet, it is reasonable to assume the beneficial effects on lipid levels were promoted through physical activity itself. Our results would support this assertion as there was no evidence of any intervention effect on total energy or changes in macronutrient content. On the other hand, although there was no intervention effect on percent body fat over the full duration of 4 years, we have reported an effect of this specialized PE to reduce percent body fat during the first 2 years of the study [12]. Therefore the possibility remains that the benefits of physical activity may have been mediated, at least in part, by a reduction in percent body fat, especially as the intervention effects on mean LDL-C in the full cohort of boys was most evident in the first 2 years.

One way a physical activity-based intervention might be expected to contribute towards lowering LDL-C is via a general increase in day-to-day physical activity, but we found no evidence of any effect on average daily pedometer step count. Instead, we suggest three other ways the intervention PE program may have been influential. Firstly, SOFIT showed there was 66% more moderate and vigorous physical activity in the intervention classes than the control classes. This finding was consistent with the final year accelerometer data showing that intervention group boys, but not girls, undertook 17% more average daily moderate physical activity during schooldays than their control group counterparts. (The gender differences in accelerometer data were also consistent with the gender difference in the intervention effect on mean LDL-C). Secondly, there was more directed fitness work in the intervention classes. However, much of this involved muscular activity associated with balance challenges and other relatively stationary exercises, which may explain the absence of any intervention effect on the running fitness test, and an absence of any pedometer-step related intervention effect. Thirdly, the intervention specialist PE classes were sustained over 4 years of elementary school. The importance of a sustained intervention over years, rather than weeks or months, is supported by a previously reported 2-year school-based physical activity intervention [34]. The latter study showed a beneficial effect on total cholesterol, although, as the authors themselves acknowledged, inferences were limited by the design of their study which involved just 2 schools.

According to the AHA and APA cutoff points for LDL-C, 20% and 16% of the 12 year-old boys and girls respectively in our cohort had elevated values. Given evidence that lipid concentrations track positively into adulthood, and that childhood cholesterol levels are linked with early vascular degeneration in adolescents [2], [4], these percentages are of concern. The beneficial intervention effect on LDL-C suggests that important health benefits can be gained from a PE program with broader educational objectives, distinguishing it from a physical training regime in which sustainability in children is questionable.

Funding is an obvious barrier to employing specialist PE teachers in many schools, and ways to better prepare classroom teachers to conduct PE are clearly of interest. To this end, as outlined in Table 1, the Bluearth Foundation provider of our PE intervention has developed an in-class professional development program for the classroom teachers, a strategy our government educational authorities may consider worthy of consideration.

Strengths of our study included the statistical model, the cluster-randomized design and the general objectivity and breadth of our data obtained from a school-based intervention consistent with school curricula objectives and already in operation in some schools. Limitations included a potential lack of sensitivity and specificity of our physical activity, fitness and dietary measurements and that our data from predominantly white children from an affluent, developed country may only be generalizable to similar populations.

## Conclusion

Well-designed specialist-delivered PE in elementary school can reduce the incidence of elevated LDL-C in elementary school-aged children and so offers an early contribution to community strategies targeting the prevention of chronic disease. Our findings are of relevance to education and health policy makers, because they show that currently adopted practices of PE as conducted by general classroom teachers do not make this level of contribution.

## Supporting Information

Checklist S1 CONSORT Checklist.
(DOCX)

Ethics S1 Ethics Application A.
(DOC)

Ethics S2 Ethics Application B.
(DOC)
