# Supplementary material for: Physical Education and Blood Lipid Concentrations in Children: The LOOK Randomized Cluster Trial
Source: PLoS One. 2013 Oct 25;8(10):e76124. doi: 10.1371/journal.pone.0076124 (PMC3808412; doi:10.1371/journal.pone.0076124)
Supplement: Ethics S1 — Ethics Application A. (DOC) [file pone.0076124.s002.doc]

**SUBMISSION TO AIS ETHICS COMMITTEE**

| **Project Title: My Child’s Health. The Lifestyle and Health of Primary School Children. A longitudinal study.**  **Part A.**  Family Influences; Academic Progress; Balance and Movement Control; Work Capacity; Influence of teacher or coach (Pedagogy); Nutrition; Measurement of Physical Activity. |
| --- |

| **Principal Researcher** | **Organisation** | **Contact details** |
| --- | --- | --- |
| Professor Richard D Telford AM PhD FACSM | Commonwealth Institute (Aust)/ANU Faculty of Medicine  Postal address: PO Box 3798  Manuka ACT 2603 | Tel: 02 62956492 (Office, Manuka) 0411237908 (Mobile)  Email:  [rtelford@cominst.org.au](mailto:rtelford@cominst.org.au) |

| **Co-workers (Section Coordinators only)** | **Organisation(s)** |
| --- | --- |
| 1. Prof. J. Carlson (Family Studies)  2. Dr L. Prosser (Academic status)  3. Mr P. Saunders (Work capacity)  4. Mr D. Stuckey (Pedagogy)  5. Ms M Martin (Nutrition)  6. Mr R M Telford (Activity)  7. 3. Mr M. McGrath (Movement) | University of Victoria.  KIDSAFE Pty Ltd, Ballarat, Victoria.  Australian Institute of Sport  University of Canberra  Private Contract Nutritionist, ACT  Commonwealth Institute  Bluearth Institute, Melbourne |

| Brief description of the project A collaborative project funded by The Commonwealth Institute, London, UK  With support and collaboration from:  The Australian Sports Commission, The Bluearth Institute, The Canberra Hospital, Australian National University, University of Canberra, Australian Institute of Sport, International Diabetes Institute, University of Sydney, Deakin University, University of Melbourne, University of NSW, Victoria University, Royal Melbourne Institute of Technology, Royal Prince Alfred Hospital, Umea University (Swe), The Cooper Clinic (USA), Australian Institute of Health and Welfare.  *A longitudinal Project with Primary School children to study the Health, Physical, Psychological, Social and Academic Development over a four-year period. Special attention will be applied to the relationship of Health and Development with movement education and physical activity, incorporating a design so that the research can continue into later life.*    One thousand children from primary schools in the ACT will be invited to this study. There will be three main testing phases, in Terms 4 2005, 2007, 2009. A variety of assessments will be performed to monitor a wide spectrum of health indicators of the children. A major thrust of the study will be to determine the relationship between physical activity and health. This will be done by (a) measuring all 1000 children’s physical activity levels and (b) 500 of the children being provided with four consecutive years of special movement education. The unique quality of this project is the multidisciplinary design bringing the collaboration of 10 distinct research groups who can share data to explain results at a level previously not explored. |
| --- |

| Detailed description of the project |
| --- |
| **Background:**    One thousand children will be invited from schools in the ACT of near average Australian socioeconomic status. An array of physical, psychological, and sociological health and development indicators will be longitudinally assessed at two year intervals. The first of these in is year 2, then year 4, year 6.  The overall aim of the study is to investigate the relationship between physical activity and health in the children. There is concern now that many of our children may not be sufficiently active to maintain good health in childhood and that this may set the scene for poor health in adulthood. Health researchers, along with the World Health Organization have requested systematic longitudinal investigation of the magnitude of the effect of physical activity on health. We have virtually no multidisciplinary longitudinal data on the effect of physical activity in the formative years of primary school on health and attitudes to health promoting behavior in adolescence and adulthood.  This study is set up with to fill this void of data by facilitating continuation into adulthood.  **The Basic Research Design**  In this first phase, we will measure 500 children beginning at age 7-8 years, prior to, midway and post a four year special program of movement education (the intervention group) and another 500 children who receive their normal school program. The movement education program will take place in the schools by experienced teachers specially trained for this purpose. Measures by the various research teams will include indicators of health in physical, psychological and biomechanical dimensions.  **The Research Hypotheses**  It is hypothesized that   1. the special Movement Education program and 2. increased volume and/or intensity of physical activity will      - facilitate an increase in physical activity and sport - improve attitudes toward physical activity and sport - stimulate improvements in bone density and geometry - reduce incidence of risk factors associated cardiovascular disease and type 2 diabetes - reduce incidence of absences from school due to sickness and disinterest - improve psychological health including self-esteem, self image, general happiness and feelings of self-worth - increase proportion of lean body mass - improve static and dynamic postural control and balance - improve the team-work and social skills - provide class-room teachers, through a professional development program, with the necessary skills to be able to conduct effective programs themselves - be more effective when the parents are involved - enhance the academic achievement of the child   Of importance at the outset of this project is the provision for a longitudinal study that spans much of the lifetime of the child. It is anticipated that data are to be collected over an extended period by a series of studies investigating selected characteristics of both experimental and control groups from age 7 to 60 years |
| **Description of subjects & recruitment process:**  The subjects are primary school children in the term 4 of year 2 (grade), aged 7-8 years. There will be 500 boys and 500 girls. They will be predominantly from Education Department schools with some private schools included. Schools will be selected after invitation to all principals to participate. Schools will be selected with certain conditions being preferred. The socioeconomic status of the districts will be such that it approximates that of average Australian cities. Schools will be preferred where composite grades are not employed. Schools will be invited in groups or clusters typical of the ACT that tend to feed into secondary schools in close proximity to facilitate the study continuing in some form in secondary school. Schools accepting the invitation must agree to a random selection of participation in the specialist movement education program. Following agreement, schools will be selected in clusters, one cluster of schools receiving the program and another cluster of schools in a socio-economically balanced area continuing with their normal physical education classes. All children will receive thorough feedback on their results through reports to the parents.  Recruitment has followed the following procedure. Firstly a discussion with the ACT Ministers for Health and Education to gain their support in principle. Secondly discussions with the Director of Curriculum in the ACT Department of Education to gain the same tacit approval. Then followed an application to do research in the ACT Department of Education. There follows meetings with the ACT school principals at the regular meeting. Finally discussions with the individual principles and letters gaining approval and acceptance by the parents, following a full description of the study and the testing procedures.  **Inclusion/exclusion criteria for participation:**  This has been explained in the previous section. Inclusion is on the basis of acceptance by the parents, teachers and principals in the schools. Exclusion is never on an individual basis as it is fully inclusive. Exclusion of a school will be on geographical/socio-economic balance in the selection process. Finally schools without composite classes in the stream beginning with grade 2 in Term 4, 2005 will be preferred.  **Methodology details:**  (including test protocols, safety procedures, copies of questionnaires etc)  **1. The effect of the family on levels of physical activity and related factors of child health.**  Parents of children in the Commonwealth Institute Project will complete the family survey on an annual basis. The instrument attached is a combination of questions of previously validated child-based family and physical activity surveys.  (a) The survey will be distributed through schools to parents and returned to the school  (b) Data will be collated, checked for missing surveys then de-identified and coded then entered into  the data base to forward for researchers to analyze and prepare reports  (c) Annual surveys will be necessary to profile family stability and habitual changes in physical  activity  **2. The relationship of physical activity levels with academic status**  Assessment of each student’s academic performance will be approached by using the results of each school’s testing of numeracy and literacy as prescribed by the Commonwealth Government’s national testing profiles. The Commonwealth requires schools in Canberra to apply such testing in grades 3, 5, 7 and 9. The academic testing takes place in term 3 of the school year during weeks 2, 3, and 4. All schools involved in the longitudinal study apply the same tests. These tests are part of existing assessments that schools already conduct. Testing protocols will strictly follow the Commonwealth Government’s national testing profiles. Data will be collated and results added to the data bank in name coded form.  **3. The relationship of physical activity and physical work capacity.**  *Assessment of physical work capacity will include two tests. Multi-stage shuttle run test – cardiovascular fitness*  Two lines 20 m apart are measured and marked. Subjects in a group of 10-15 start on one of the lines, and run towards the opposite 20m line, aiming to reach it and turn as the next beep is emitted from the CD (single beep).. Subjects continue running for as long as possible, until they are unable to maintain the speed as indicated by the CD. The end of the test is indicated by the inability of a subject to maintain the required pace for two successive beeps.  *Vertical jump test – Muscle strength and power*  The Vertec© apparatus is based on the principle of displaceable plastic vanes that record jump height. Standing Reach Height is measured by extending the arm fully above the head and maximum number of vanes is displaced whilst feet are maintained flat on ground. Subjects then use a countermovement and jump upwards with the arm extended and touch as many vanes as possible, three attempts are given. Vertical jump is recorded by subtracting standing height from jump height.  **4. Pedagogy.**  The effective of teacher/instructor styles on health outcomes of the children.  Participating Teachers, Parents and Students will be identified, recruited and their permission elicited as part of the larger project Methods of data collection Teacher questionnaire, Parent and Student Questionnaires (as attached) will be distributed through the school with a self-addressed envelope for return. Observational Checklists will be filled out at the participating schools along with Case Study information by University Staff and selected students trained and paid for their services.  **5. Nutrition.**  Subject’s parents and teachers will be asked to assist filling in a one day dietary record describing what the child consumed for that given day. The dietary records used will be a modified version of dietary records adopted by most clinicians and is similar to the records used at the AIS. Parents and teachers will be given instructions how to complete the dietary record and also given guidance on estimating portion sizes of foods consumed. Once the record has been completed that parents will return them to the teachers in a sealed envelope to maintain confidentiality. Any queries will be followed up with a phone interview to the parents to confirm the record is completed accurately.    *Coding of Data:*  Each food reported on the records will be checked to confirm that the weight (amount) and food description is documented. For unique foods a dietician will research food labels, preparation techniques, ingredients and packaging in grocery stores and at the school canteen to ensure accuracy.  *Processing Data:*  Data for each child will be entered by an experienced coder into Australian compatible food database (e.g., “Food Works” )After each record is entered into the database a food by food evaluation will be performed to ensure accuracy of data. During data processing one copy of the records will be stored and locked up in files at 5 Waine Place Nicholls ACT 2913 and the other copies will be stored at the ASC.  **6. Measurement of Physical Activity**  Physical activity levels will be measured in all children in term 4 each year for the duration of the study. Two methods of measurement will be used.    *1) Questionnaire*  A proxy questionnaire will be administered to parents and will report their child’s physical activity levels for a typical week. The questionnaire will be a modification of the CLASS questionnaire  *2) Motion Sensor* (accelerometry or pedometry )  Each child (n=1000) will wear a triaxial accelerometer or pedometer for a period of 5 consecutive days. Testing will be staggered with 100 children being tested each week over a 10 week period (term  Each testing week will commence on a Wednesday and will consist of 5 days wearing an accelerometer (Wednesday to Sunday) and two days of data collection and preparation for the following group (Monday and Tuesday).  7. **Markers of Motor Control Performance**  The section involves specific measurements, referenced in the international literature, to evaluate movement strategies, movement skill, balance and muscular endurance.   - Picking up a coin from the floor   To examine the strategy used by the subject without their knowledge in this simple but highly functional activity. The style of the lift can be categorized from video observation into five levels, with one extreme being a full squat and the other extreme being a straight legged bend.   - Wobble Board Balance   A dynamic balance test, using a wobble board can provide data on the efficiency of balancing during a difficult standing task. Measurement of the dynamic balance skill is by simply counting the number of times the rim of the board hits the floor and a description of the stabilizing strategies employed.   - Full Body Balance   Following the same principal as the wobble board test, a full body balance test can be constructed, using a full body balance board. The child is asked to balance in a quadruped and then a supine posture for 30 seconds. The number of times the board hits the floor is measured.   - Slow Stepping   Slow stepping is a method of examining the pattern of locomotion efficiency. Stepping will be performed on a force plate. Scoring is based on observation of the technique and force plate analysis.   - Movement Repositioning test.   The subject performs stepping from a fixed point, and the distance and amount of rotation is measured from the fixed point. Eyes open looking at the horizon.  Eyes closed.   - Endurance Testing of the lumbar spine in three planes of movement. The subject, in a lying position is asked to hold various postures, the holding time recorded.   In extension using the Beiring-Sorensen position (Beiring-Sorensen 1984)  In flexion using a sit up posture  In right and left lateral bending in a side bridge posture  (BIERING-SORENSEN, F. Physical measurements as risk indicators for low-back trouble over a one-year period, Spine, 9, 106 – 119. 1984).   - The Rod and Frame Test (RFT) (a test of perceptuo-motor performance)   The RFT first described by Wilkins, uses an illuminated tilted frame with an illuminate rod embedded within it. The subject is in a darkened room and is asked to judge vertical. This test is used to test what is known as field dependence or independence. (The field dependent individual (FD) cannot ignore the ambiguous information from the frame and align the rod to the tilted frame).  **Method of data analysis:**  There will be 500 students in a control group, and 500 in an experimental group that receives a specialized movement education program. For both groups, a wide range of data will be gathered on variables including the level and intensity of physical activity, and a wide range of health and education outcomes.  The study will generate a uniquely rich information base that will support analyses of the relationships between physical education/activity and key outcomes for children. In particular, it will support:   1. Contrast analyses between those who participate and do not participate in the program of physical education -- the differences between the two groups' patterns of physical activity and the groups' health and education outcomes. 2. For both groups, analyses of the relationship between the volume and intensity of physical activity and key outcomes. 3. For both groups, analyses of the complex inter-relationships between the multiple dimensions of risk/protective factors and outcomes. This is a particularly important feature of this study, in contrast to other studies that have analysed the relationships between just one or a few factors and just one or a few outcomes.   **Proposed time-frame:**  The project will begin in Term 4, 2005, October 10. The first major data collection period will  be completed at the end of Term 4, on December 16. The second and third (final) data collection periods will be in Term 4, 2007 and Term 4, 2009. Final reports and papers will be written in 2010.  **Budget required: (for Parts A and B combined)**  $ 3.5 million  Approved level of funding: $ 3 million plus a similar amount in kind from universities and other institutions. Source of funding: Commonwealth Institute, London, UK . Signed off at CHOGM .  **Describe how you will maintain security/anonymity of participants:**   - Only the primary investigators in each section/project will have access to the results in their section. - All results will be stored electronically in a central data bank using ID number codes for each subject. This central data bank will be under password protection. - Only the Research Director, Data Manager, and Research Section Coordinator will have access to the coding system. - Only the Research Director will have access to material from more than one project team. Only upon agreement at meetings between the coordinators of each section will data be shared and even then it will be dissipated in numerical form, no names associated. - Results will not be forwarded to any third parties without written permission from parents, project director and section coordinator. - One hard copy of all raw and collated data will be held by the Section Coordinator in question in a fully secured cabinet. - No names or means of identifying subjects will be included in any scientific manuscripts prepared for peer-reviewed scientific or medical publications. - All subjects recruited into the study will be assigned a study code ID number.  Only the Data Manager and the Principal Researcher will have the key to unlock the study code numbers.   All researchers and their institutions will enter into a research contract with the Commonwealth Institute which binds them to subject confidentiality and duty of care agreements. |
| **What will be gained by undertaking the research?**  The enormity of the health problems resulting from the societal drift to more sedentary lifestyles is becoming increasingly public. Recognised authorities such as the World Health Organization (WHO) have published extensively on the severe risks associated with growing global levels of physical inactivity.  The impact of physical inactivity is being felt in the increasing incidence of Type II Diabetes, cardiovascular disease, skeletal disease such as osteoporosis and some forms of cancer such as colorectal, breast, and prostate. Physical inactivity has also been associated with the development of psychological problems.  In 2000, the Commonwealth Department of Health and Aged Care and the Australian Sports Commission undertook a study to investigate the impact of physical inactivity of Australians, attributing 23,500 deaths, 845,000 hospital admissions, and costs of up to $5.6 billion to the problem each year (Stephenson et al, “The Cost of Illness Attributable to Physical Inactivity” - 2000).  Numerous reports have highlighted the increasing levels of obesity and inactivity in children, not only in Australia, but world-wide. Signs of onset of preventable disease are now occurring in our children. Indeed several studies have alluded to early signs of cardiovascular and metabolic dysfunction in primary school aged children.    This study will contribute to the medical scientific literature by investigating the impact of physical activity (and inactivity) in the formative years of primary school on health of our children, paving the way for the investigation to continue into adolescence and adulthood. This study will be unique in the breadth of its multidisciplinary approach.  Reports will be presented to relevant state and national government decision-makers and the population in general. The basis of such reports will be the role of physical activity in general, and physical education in schools or after-school programs, on the health and well-being of our children. Reports will also be presented by the Commonwealth Institute to Commonwealth Heads of Government Meeting (CHOGM). |

| **How will the results of the study be presented?**  The Research Contracts between the Commonwealth Institute and each Research Section include agreements for main reports to be written in each of the three years of major data collection, 2005, 2007, and 2009. The titles of these reports have been pre-determined and centre around the effect of physical activity and movement education in schools on the particular health indicators measured by each group. Furthermore, meetings between collaborators are to be held to determine the priority order of importance of papers to be presented to international peer-reviewed journals and conferences. In general, lay reports for the public, through the media, will be released at that time of publication following the peer review system. |
| --- |

Have you provided a copy of the ***Informed Consent***? Yes

Have you provided a copy of the ‘***Information to Participants***’? Yes

Have you included and signed the ‘***Clearance Requirements***’ questionnaire? Yes

Have you included and signed the ‘***Privacy Principles***’ agreement? Yes

Has ***funding*** for this project been approved? Yes

Signed: …………………………………………………………… / /

Principal Researcher Date

**CLEARANCE REQUIREMENTS**

1. Where a proposal involves the participation of human subjects, approval must be obtained from the AIS Ethics Committee to determine whether or not the project is acceptable on ethical grounds.

Does this project involve the participation of human subjects? **Yes**

If YES please complete the following:

ETHICAL IMPLICATIONS OF THE PROJECT

2 Does this section of the proposal involve human subjects participating in:

(i) Biomedical procedures? **No**

(ii) procedures to elicit information through personality tests, questionnaires, inventories, surveys, observations? **No**

(iii) procedures specifically designed to directly modify the knowledge, thinking,

attitudes, feelings or other aspects of the behaviour of subjects? **No**

3. If biomedical procedures are involved: **N/A**

(i) Are you qualified/certified to perform the procedures? **N/A**

(ii) If you are not qualified/certified to perform the procedures, has a qualified

medical practitioner approved of the biomedical procedures? **N/A**

(iii) will this study involve drugs or chemical agents (dosages), ionising radiation, non-ionising radiation (microwaves, lasers), or high intensity sound? **N/A**

4. Does this study involve giving false or misleading information to subjects or withholding information from them such that their “informed” consent is in question? **No**

5. Are the procedures to be used new or innovative (not established and accepted)? **No**

6. Will the procedures cause any degree of discomfort, harassment, invasion of privacy,

risk of physical injury, threat to dignity of subjects or be otherwise potentially harmful

to subjects?

**Yes there will be a degree of discomfort associated with leg and respiratory fatigue when the children carry out the multistage run as they must run until they cannot keep up with the CD “beep”. This test will be repeated each two years.**

If YES, have specific provisions been made to correct any harmful or adverse conditions that may arise?

**Two aspects relate to this test. The first is that as soon as the child does not make the required marker in time with the “beep” of the CD, he or she will be asked to stop. This removes much of the discomfort of continuing when in a fatigued state. The second aspect is making sure that the child is not embarrassed or de-motivated with a poor performance. Encouragement and congratulatory remarks will be given by the testing team to each child. Children will be grouped in small groups of 4 or 5 so that children of similar standards can be tested together. This test conducted in this way is enjoyed by the children who inevitably urge each other on.**

1. How do the benefits outweigh the risks? (**give a brief description**)

**The risks of falling or succumbing to a muscular injury in the assessments are not high. Benefits of the knowledge of the fitness or movement capability as important potential explanators of health risks outweigh any risks.**

8. Will any type of electrical equipment be used that will be connected to subjects, and if so please indicate which category the equipment belongs? **No**

9. Will subjects receive any monetary or other benefits for participating? **No**

Are any manufacturers and/or suppliers of products contributing financially to the project? **No**

If yes, attach details

10. Does the project involve subjects who are:

(i) scholarship holders? **N/A**

(ii) minors (i.e. less than 18 years of age)? **Yes**

(iii) mentally disabled? **No**

(iv) physically disabled? **No**

11. Do procedures include obtaining parental or guardian consent for access to subjects if they are minors (i.e. under 16 years) or mentally disabled? **YES**

12. Are procedures for maintaining confidentiality of all subjects’ data fully described? **YES**

13. Are procedures for obtaining informed consent fully described? **YES**

14. Is there a copy of the informed consent document (signed document with explanation

of the study, transmittal letter, debriefing statement, or other) to each subject attached? **YES**

15. Give estimates for:

(i) average time required for subject’s participation (in hours)

**Times are estimates per test session (every two years) unless otherwise indicated**

**1. Family Studies Parents 30min yearly**

**2. Academic status None**

**3. Movement Child 1 hour**

**4. Work capacity Child 30 min**

**5. Pedagogy Parent 30 min Child 30 min yearly**

**7. Nutrition Parents 1 hour Child 30 min yearly**

**8. Activity Child 30 min explanation time.**

(ii) the total number of items if questionnaires/tests are involved 4

(iii) the number of volunteers (subjects) to be involved with this study 1000

16. Has funding been approved for this project? **YES**

17. Has the proposal been endorsement by the appropriate AIS Head Coach. **N/A**

Attach letter of endorsement (*not required if State Institute/Academy athletes & staff*)

18. Has the proposal been endorsed by the appropriate AIS Head of Department? **N/A**

CERTIFICATE OF PRINCIPAL RESEARCHER

19. The project or activity described in this application was planned in accordance with the National Health and Medical Research Council’s *National Statement on Ethical Conduct in Research Involving Humans*. I understand that any change in the protocol of the research project, either before or during the project, which alters the project from that described in this application and the project being undertaken. Further, I undertake to notify the Ethics Committee of any unanticipated events that do or could affect the safety and well-being of subjects. I will also inform the Committee of the completion of the study and the way in which the outcomes are communicated. In the event that the project does not commence or is terminated, I will inform the Committee through its Secretary.

____________________________________ ____________

Signature Date

SIGNATURE OF RESEARCHERS

20. Have each researcher listed in response to Question 3, sign here

APPLICABLE ONLY IN SECTION B

# INFORMATION ON ‘INFORMED CONSENT’

The researcher is required to protect the rights and well-being of subjects in his or her study and obtain informed consent. The basic elements of informed consent are as follows:

The following summary (simplified further if required) will be provided to teachers and parents

2. (a) **Project Title and Brief Description.**

**My Child’s Health. The Lifestyle and Health of Primary School Children.**

A Longitudinal study of the children’s health with special reference to the relationships between lifestyle factors and risk factor for non-communicable health problems ( including cardiovascular disease, type 2 diabetes, bone health, balance and motor control, anxiety and self-esteem, immunological status)

(b) **Preliminary note on the study**

This study is funded by the Commonwealth Institute, London UK operating on behalf of Commonwealth Nations, and various universities and including The Canberra Hospital, ANU, Uni of Syd, Uni of Melb, RMIT, Victoria Uni, Uni of NSW, Canberra Uni, Int Diabetes Inst, Umea Uni (Sweden), and the Aust Sports Commission.

The ACT has been chosen internationally as the preferred venue to carry out this research. Arguments put forward to the Commonwealth Institute in securing Canberra as the preferred venue included its ideal sized population with a greater than average interest in education and research, its self-government, and the progressive nature of its school system.

3. **Project Director**

Surname: **Telford**  Given Names:  **Professor** **Richard D.**

Address: **PO Box 3798 Manuka ACT 2603**

5. **Key Investigators and their Organisations**

Prof .David Celermajer Cardiovascular Health Medical Faculty, Univ. of Sydney

Prof. Roland Stocker Cardiovascular Health Medical Faculty, Univ. of NSW

Dr. Paul Waring Cardiovascular Health Dept of Chemistry, ANU

Prof. Marc Budge Cardiovascular Health Faculty of Medicine, ANU

Prof. Paul Zimmet Metabolism Function (Type II Diabetes) International Diabetes Institute

Dr. Jonathan Shaw Metabolism Function (Type II Diabetes) International Diabetes Institute

Dr. David Dunstan Metabolism Function (Type II Diabetes) International Diabetes Institute

Prof. Mark Hargraves Metabolism Function (Type II Diabetes) Medical Faculty, Univ. of Melb.

Prof. John Hawley Metabolism Function (Type II Diabetes) Faculty of Applied Science, RMIT

Prof. Shona Bass Skeletal Health Health Sciences, Deakin Univ.

Dr .Rob Daly Skeletal Health/Family Influence Health Sciences, Deakin Univ.

Dr. Geraldine Naughton Skeletal Health Fac. Sci, Catholic Univ, NSW.

Prof. Markus Seibel Skeletal Health Medical Faculty, Univ. of Sydney

Dr. Jeff Symons Balance, Movement, Skill Bluearth Institute, Melbourne

Mr. Mark McGrath Balance, Movement, Skill Bluearth Institute, Melbourne

Mr. Wayne Haynes Balance, Movement, Skill Bluearth Institute, Melbourne

Prof. John Carlson Family and Community Influence Faculty of Appl Science Vic. Univ.

Mr Rohan Telford Physical Activity/Fitness Commonwealth Institute

Dr Philo Saunders Physical Activity / Fitness Physiology Dept, AIS

Ms Mary Martin Nutrition Private Nutritionist, Canberra

Prof. Don Byrne Psychology Dept of Psychology, ANU.

Dr. Laurie Prosser Psychology / Academic Success K.I.D.S. Ballarat

Dr. Xiaoli Jiang Psychology Dept of Phys.Ed. Ballarat Uni.

Dr. David Pyne Immunological Competence Dept of Physiology, AIS

Dr. Ken Tallis Academic Success, Statistics Aust. Inst of Health and Welfare

Mr. Darryl Stuckey Pedagogy Faculty of Education, Canberra Univ.

Dr Robert Fitzgerald Pedagogy Fac. Education, Canberra Univ.

Prof. Dennis Goodram Pedagogy Fac.of Education, Canberra Univ.

6. Brief description of the **major objectives** of the project:

**(1)** To investigate the impact of lifestyle and other environmental influences on the physical and psychological well-being and development of children in primary school children

**(2)** To investigate the effectiveness, and the class-room teacher sustainability, of a movement education program delivered by specialist movement educationists.

**(3)** To design tests such as to provide an opportunity for ongoing health monitoring through adolescence and adulthood to old age.

1. Brief description of the project’s proposed **methodology**:

(1) **Numbers of Children and Timing** We will monitor 1000 children beginning at age 7-8 years (grade 2) in Term 4, 2005, again in 2007, and finally in Term 4, 2009 during a four-year period. Our aim is to investigate the impact of environmental factors including physical activity, nutrition, family and school on their physical, psychological and sociological health, growth and development.

(2) **The Movement Education program** In particular we will provide specially trained physical educators to deliver a carefully designed movement education program twice a week to 500 of the children for the four years. Whilst all of the 1000 children will be monitored in terms of their health, nutritional intake and overall physical activity in and out of school, we will be able to monitor any further impact of the special movement education. A feature of the movement education program is that it aims to foster a love of physical activity in every child, irrespective of body build or coordination, in an absolutely non-threatening and “fun” environment.

(3) **The Interdisciplinary design** The uniqueness and significance of this study is due to its interdisciplinary nature. A wide array of tests will be carried out including those indicating early signs of cardiovascular health, bone health, psychological health, as well as aerobic fitness and muscular power, body composition, levels of physical activity and nutritional intake. The interdisciplinary design and subsequent array of potential cofactors in the statistical analyses will enable researchers to draw more meaningful conclusions, especially in terms of causal relationships.

(4) **Venues for the measurements** One of these tests will require an excursion to The Canberra Hospital where measures of bone density and geometry will be carried out. All of the remaining tests will be carried out at the school, usually with one or two students required at a time. **Where children are measured as a class, such as fitness measures, it is intended that we will use part of the time allocated to the physical education curriculum.**

(5) **Integration with the class-room teacher and curriculum** Each of the major tests, such as those relating to heart function, bone health, nutrition, physical activity measures, balance measures etc will be accompanied by notes, diagrams and information that the class-room teacher may wish to incorporate into the health and physical education curriculum. The research team hopes that many aspects of this project will serve as an educational experience with researchers working in close liaison with the class-room teacher.

(6) **Reports to Parents (and teachers)** During the course of the study, teachers, students and parents will receive easily understood reports summarising the health and growth of the children. All results will be absolutely confidential and extreme care will be taken to prevent any form of embarrassment or undue concern to the children. In fact, to the contrary and consistent with the movement education program, we intend to make the experience an enjoyable one for all.

(7) **Nature of the Tests – Summary (This list included Parts A and B of the project)** It is our intention that the children will find the tests interesting and enjoyable. The tests are as follows:

(a) An ultrasound procedure where this non-invasive method produces images of the heart and blood vessels Time required 30 min per child, 2 children at a time from class. Venue: school

(b) Two scans for bone density and geometry and body composition (at The Canberra Hospital). These are routinely performed **with children and involve extremely low exposure to X-ray (standard techniques have already met Ethics Committee approval)

Time required 30 min per child test time plus travel to hospital. Total time 2 hr excursion for 4 children at a time.

(c) One venous blood sample* for indication of risk of cardiovascular disease, type 2 diabetes, and nutritional deficiency. Time required 20 min per child, before school if possible. Venue: school. Breakfast funded by Project, from school canteen

(d) Wearing of an accelerometer or pedometer for 5 days at home and school to assess amount of physical activity. Time required 20 min per class to explain how to wear the accelerometer. A questionnaire to parents will be used in conjunction with the accelerometers. Venue: school, physical education class

(e) Two simple motor performance tests, a vertical jump test and a test of aerobic capacity. Time required 20 min Venue: school, physical education class

(f) A group of balance and coordination tests, aimed at measuring posture during movement. Time required 30 min Venue: school, physical education class

(g) A questionnaire (to children) to assess psychological components of health and development (including self-esteem, anxiety, self-image) and family influences on physical activity. Time required 30 min Venue: school

(h) A diet diary will be provided (to parents) to assess nutritional intake. There will also be an interview with parent and child. Time required 1 hour per family to complete questionnaire and do interview. Venue: school or home

(i) To assess the teaching effectiveness the visiting teachers involved in delivery of the specialist movement education programs will be interviewed during the course of the year. Class-room teachers will be interviewed and asked to fill in a questionnaire in regard to their assessment of the movement education program. There will be further investigation of the effectiveness of the professional development program for the class-room teacher and this will be assessed with observation of the class and interviews with the movement specialists, children and teachers themselves. The pedagogical research team seeks to utilize videotaping of movement education classes to investigate the styles of teaching. Extreme care will be taken with the confidentiality of the videotape. As for other test areas, it is fully understood that permission must be obtained from parents and teachers and the children. Time required 30 min. Venue: school physical education classes

*A note on blood sampling. Taking a blood sample is the only measure that involves discomfort. A blood sample is required at the beginning, at two years and again at four years into the study. Unfortunately we cannot avoid examination of blood if we are to effectively and carefully monitor health of the heart, blood vessels, and metabolism. To minimize discomfort we have limited this measure to once every two years and the sampling will be carried out by experienced phlebotomists. All of our researchers are highly experienced in their respective areas, well accustomed to taking the special care necessary in working with children.

** Notes on the bone and body composition measures. These involve the use of a two scanning machines, one for who body mineral content of the bone , and body muscle and fat tissue. The other is specific to the leg region where bone structure is scanned for shape and density.

These machines are new machines, designed to minimize radiation. “DXA scanning provide a safe means which to assess children and adults for single and repeated measures…..A single DXA examination is equivalent to about 0.03% of the natural annual dose of radiation (due to general living on earth) ……This is less than 1/100^th^ of the radiation of a chest X-ray” (from “Physical Activity and Bone Health” Khan et al Human Kinetics (publ) 2001).

And from the experienced research group from Deakin University/Sydney Universtiy (represented by Associate Professor Shona Bass and Professor Marcus Seibel)

“This research study involves exposure to a very small amount of radiation. As part of everyday living, everyone is exposed to naturally occurring background radiation and receives a dose of about 2 millisieverts (mSV) each year. The effective dose from this study is about 0.0135 mSV. At this dose level, no harmful effects of radiation have been demonstrated as any effect is too small to measure. The risk is believed to be minimal”

(8) **Duty of Care** Any child found with a measure suggesting immediate medical attention will be advised in a letter to the school or their parents as required, as part of the health report.

(9) **Ethics Committee Approval** All procedures will have been examined by Ethics Committees either at the Australian Institute of Sport or at the University involved. These committees are highly experienced in ensuring that all research complies with the highest standards of safety, care, purpose and confidentiality.

8  **Which schools do you intend to ask to participate in the project?**

Approximately 25 schools in the ACT are required to obtain 1000 children in Grade 2. Ideally there would be a random selection of schools, but this is not possible. Schools will be invited to participate based on their geographical location and indication of the willingness of principals to be involved. Further factors to be considered include our preference for schools with the more stable enrolments, and for schools not employing composite classes.

9**. Total time required** of schools to participate in the research (this includes all activities).

(a) Approximate test time per child: 4 hr in year 2005, 2007, and 2009. In addition there will be 2 questionnaires administered in the “non-assessment” years along with measures of height and weight. Time: 1hr. In most cases tests will be administered to two or three children at a time, at the school. Children will be requested from class for the 30 min period that is generally required. Alternatively, some tests involving activity are anticipated to be performed during physical education classes.

(b) Approximate time for questionnaires/interviews per child: 1.5 hr and per teacher: 2 hr

10 **Please provide a planned schedule specifying the date when the major steps in your project will be started and completed**

**Step** **Test date**

*Measurement period 1* Term 4 2005

Testing of 1000 children will be

completed in Term 4.

*Measurement period 2* Term 4 2007

Measurement period 3 Term 4 2009

Project completed end Term 4

There will be some tests, such as selected questionnaires, height, weight performed each year.

**PARENTAL CONSENT FORM**

Project Title: My Child’s Health. A longitudinal study of the relationship between lifestyle and health of primary school children.

Project Director: Richard D. Telford AM PhD FACSM

This is to certify that I, ________________________________ hereby agree to (give permission to have my child) participate as a volunteer in a scientific investigation as an authorised part of the research program of the Australian Sports Commission under the supervision of _____________________________.

The investigation and my (child’s) part in the investigation have been defined and fully explained to me and I understand the explanation. A copy of the procedures of this investigation and a description of any risks and discomforts has been provided to me and has been discussed in detail with me.

- I have been given an opportunity to ask whatever questions I may have had and all such questions and inquiries have been answered to my satisfaction.
- I understand that I am free to deny any answers to specific items or questions in interviews or questionnaires.
- I understand that I am (my child is) free to withdraw consent and to discontinue participation in the project or activity at any time.
- I understand that any data or answers to questions will remain confidential with regard to my (child’s) identity.
- I certify to the best of my knowledge and belief, I have (and my child has) no physical or mental illness or weakness that would increase the risk to me (him/her) of participating in this investigation.
- I am (the child is) participating in this project of my (his/her) own free will and I have (the child has) not been coerced in any way to participate.

Signature of Subject: _______________________________ Date: ___/___/___

Signature of Parent or

Guardian of minor: (under 16 years) ______________________________ Date: ___/___/___

I, the undersigned, was present when the study was explained to the subject/s in detail and to the best of my knowledge and belief it was understood.

Signature of Researcher: _____________________________ Date: ___/___/___

**THE INFORMATION PRIVACY PRINCIPLES RELATING TO RESEARCH**

**Guidelines for the protection of privacy in the conduct of medical research.**

The written protocol for the conduct of each medical research project shall state the aims of the study, the data needed, the source of the data, the reasons why personal information is needed and the way in which the data will be collected, disclosed. used and protected. The protocol should state:

(a) the specific uses to which the personal information acquired or developed during the study will be put;

(b) the estimated time of retention of the personal information (Good scientific practice entails the retention of data for periods of at least five years[NHMRC Statement on Scientific Practice]);

(c) security procedures to be applied to the personal information;

(d) that data will be retained in accordance with good scientific practice and in a form that is at least as secure as it was in the sources from which the data were obtained;

(e) the safeguards that will be applied to protect personal information that will be made available to other researchers or third parties;

(f) a list of personnel with access to the personal information;

(g) proposed methods of disposal of the personal information having regard to the Archives Act, 1983 for Commonwealth records and the legislative requirements of a State or Territory of the Commonwealth;

(h) whether and in what form, the final results will be published or made available to other researchers;

(i) what will be done with personal information on the completion of the study;

(j) the safeguards that will be applied to protect personal information, disclosed by a Commonwealth agency, including the terms of a disclosure agreement which may be entered into the between the agency and the researcher, governing limits on use and disclosure of personal information to obtained from the agency.

***INFORMATION PRIVACY PRINCIPLES IN PLAIN ENGLISH***

**Principle 1** - **Restricting collection of information to lawful purposes and by fair means.**

Agencies must not collect personal information unless (i) it is collected for a lawful purpose

directly related to a function the agency; and (ii) the means of' collection are lawful and fair.

**Principle 2 - Informing people why information is collected.**

Agencies must ensure that people from whom they solicit personal information are generally aware, before collection, or as soon as practical thereafter, of (i) the purpose of collection; (ii) any legal authority for the collection; and (iii) any third parties to which the collecting agency discloses such information as a usual practice.

**Principle 3 - Ensuring personal information collected is of good quality and not too intrusive.**

Where an agency solicits personal information (whether from the person that the information is about or otherwise), it must take reasonable steps to ensure (i) that the information is relevant to the purpose of collection, up-to-date and complete; and (ii) that its collection does not unreasonably intrude upon the person's personal affairs.

**Principle 4 - Ensuring proper security of personal information**

An agency must protect personal information against misuse by reasonable security safeguards, including doing everything within its power to ensure that authorised recipients of the information do not misuse it.

**Principle 5 - Allowing people to know what personal information is collected and why.**

Any person has a right to know whether an agency holds any personal information (whether on him or her or not), and if so (a) its nature; (b) the main purposes for which it is used; (c) the classes of persons about whom it is kept; (d) the period for which each type of record is kept; (e) the persons who are entitled to have access to it, and under what conditions; and (f) how to obtain access to it. Each agency must maintain a register of this information and must inform the Privacy Commissioner annually of its contents.

**Principle 6 - Allowing people access to their own records.**

A person has a right of access to personal information held by an agency, subject to exceptions provided in the Freedom of Information Act 1982 or any other law.

**Principle 7 - Ensuring that personal information stored is of good quality, including allowing people to obtain corrections where it is not.**

Agencies must make corrections, deletions and additions to personal information to ensure that it is (i) accurate; and (ii) relevant, up-to-date, complete and not misleading (given the purpose of collection and related purposes), subject to exceptions provided in the Freedom of Information Act 1992 or any other law. Agencies are also required to add a reasonable statement by a person to that person's record, on request.

**Principle 8 - Ensuring that personal information is of good quality before using it.** Agencies must take reasonable steps to ensure that personal information is accurate, up-to-date and complete (given the purpose of collection and related purposes) before using it.

**Principle 9 - Ensuring that Personal information is relevant before using it.**

Agencies may only use personal information for purposes to which it is relevant.

**Principle 10 - Limiting the use of personal information to the purposes for which it was collected.**

Agencies may not use personal information for purposes other than for which it was collected except (a) with the consent of the person; (b) to prevent a serious and imminent threat to a person's life or health; (c) as required or authorised by law; (d) where reasonably necessary for the enforcement of criminal or revenue laws; or (e) for a directly related purpose in the case of exception (d), but not otherwise, the use must be logged.

**Principle 11 - Preventing the disclosure of personal information outside the agency.**

Agencies may not disclose to anyone else personal information, with the same exceptions as apply to Principle 10 (a) - (d), plus an additional exception where the subject of the information is reasonably likely to be aware of the practice of disclosure (or reasonably likely to have been made aware under Principle 2). The recipient of information under one of these exceptions may only use it for the purpose for which it was disclosed.

I ........................................................................................................................ acknowledge the Information Privacy Principles and undertake to ensure that these principles are adhered to with reference to collection of data from subjects, for the purpose of this research project.

Signed ............................................................................. Project Director

Date .........................................

APPENDIX QUESTIONNAIRES/OTHER FORMS USED IN THIS STUDY

1. FAMILY INFLUENCES

# PARENT / GUARDIAN SURVEY

**SECTION ONE: FAMILY PROFILE**

(PLEASE NOTE: This information will be held in strict confidence and will only be accessed by the research team).

| Name of your child: |  | | | | | | | | | | | | | | | | | | | Date: | | | |  | | | | | | |
| --- | --- | --- | --- | --- | --- | --- | --- | --- | --- | --- | --- | --- | --- | --- | --- | --- | --- | --- | --- | --- | --- | --- | --- | --- | --- | --- | --- | --- | --- | --- |
| School your child attends: | |  |  | |  | |  | | |  | |  | |  | |  | | |  | |  |  |  |  |  | |  | |  | |
| Town/Suburb in which you live: | | | |  | |  | |  |  | |  | |  | |  | |  | Home postcode: | | | | | | | |  |  |  | |  |

How long have you lived in your local area?

⬜ _____ Years

- **Don’t know**

The following questions will help us to find out more about the *parents* of children in Commonwealth Institute Project.

1. Are you:

⬜ Male ⬜ Female

1. Age category:

**Self Spouse/Partner**
⬜ 20-25 years ⬜ 20-25 years
⬜ 26-30 years ⬜ 26-30 years

⬜ 31-35 years ⬜ 31-35 years

⬜ 36-40 years ⬜ 36-40 years
 ⬜ 41-49 years ⬜ 41-49 years

⬜ 50 + years ⬜ 50 + years

1. Main occupation?

**Self Spouse/Partner**

__________________________ __________________________

1. Which of the following best describes YOUR current employment?
   (tick **one** box)

**Self Spouse/Partner**
⬜ Employed/self employed ⬜ Employed/self employed

Full time Full time

⬜ Employed/self employed ⬜ Employed/self employed

Part time Part time

⬜ Home duties ⬜ Home duties

⬜ Pensioner ⬜ Pensioner

⬜ Other ⬜ Other

1. Highest level of education completed?

**Self Spouse/Partner**

⬜ Primary school ⬜ Primary school

⬜ Year 7/8 ⬜ Year 7/8

⬜ Year 9/10 ⬜ Year 9/10

⬜ Year 11/12 ⬜ Year 11/12
⬜ Trade apprenticeship ⬜ Trade apprenticeship
⬜ Technical diploma/certificate ⬜ Technical diploma/certificate

⬜ Tertiary degree ⬜ Tertiary degree

⬜ Post graduate degree ⬜ Post graduate degree
⬜ Other ________________ ⬜ Other _________________

1. Which of the following best describes your current marital status?

| ⬜ | ⬜ | ⬜ | ⬜ | ⬜ |
| --- | --- | --- | --- | --- |
| Single/Never married | Married | Defacto/ partner | Separated/ Divorced | Spouse  deceased |

1. Country of birth?

**Child’s mother** **Child’s father**

⬜ Australia ⬜ Australia
⬜ Other country (please name) ⬜ Other country (please name)
 ______________________ ______________________
⬜ Don’t know ⬜ Don’t know

1. Is the child’s MOTHER of Aboriginal or Torres Strait Islander origin?

⬜ Aboriginal but not Torres Strait Islander

⬜ Torres Strait Islander but not Aboriginal origin

⬜ Aboriginal and Torres Strait Islander origin

⬜ Not Aboriginal or Torres Strait Islander origin

⬜ Don’t know

1. Is the child’s FATHER of Aboriginal or Torres Strait Islander origin?

⬜ Aboriginal but not Torres Strait Islander

⬜ Torres Strait Islander but not Aboriginal origin

⬜ Aboriginal and Torres Strait Islander origin

⬜ Not Aboriginal or Torres Strait Islander origin

⬜ Don’t know

**The following questions refer to your child**

1. Which of the following best describes your relationship to this child?

| ⬜ | ⬜ | ⬜ | ⬜ | ⬜ |
| --- | --- | --- | --- | --- |
| Biological parent | Step parent | Guardian/ Foster parent | Adoptive parent | Other (please explain below) |

# Who does your child usually live with?(tick more than one box if appropriate)

| Mother | ⬜ | Brother(s) | ⬜ |
| --- | --- | --- | --- |
| Father | ⬜ | Sister(s) | ⬜ |
| Stepmother | ⬜ | Stepbrother(s) | ⬜ |
| Stepfather | ⬜ | Stepsisters(s) | ⬜ |
| Grandparent/s | ⬜ | Other (please specify below) | ⬜ |

1. What is your child’s date of birth?

____ / ____ / ____

Day Month Year

What country was your child born in?

⬜ Australia
⬜ Other country (please name) _______________________

⬜ Don’t know

1. Is your child of Aboriginal or Torres Strait Islander origin?

⬜ Aboriginal but not Torres Strait Islander

⬜ Torres Strait Islander but not Aboriginal origin

⬜ Aboriginal and Torres Strait Islander origin

⬜ Not Aboriginal or Torres Strait Islander origin

⬜ Don’t know

1. Is your child:

⬜ Male ⬜ Female

16. This child is the _______ (tick **one** box) in the family

| ⬜ | ⬜ | ⬜ | ⬜ | ⬜ |
| --- | --- | --- | --- | --- |
| 1^st^ child | 2^nd^ child | 3^rd^ child | 4^th^ child | 5^th^ child or more |

1. How many children are in the family altogether? (tick **one** box)

| ⬜ | ⬜ | ⬜ | ⬜ | ⬜ |
| --- | --- | --- | --- | --- |
| 1 child | 2 children | 3 children | 4 children | 5 children or more |

18. Are you concerned about your child’s height? No ⬜ Yes ⬜

If yes, what are your concerns ________________________________________

19. Are you concerned about your child’s weight? No ⬜ Yes ⬜

If yes, what are your concerns ________________________________________

________________________________________________________________

20. Do you have a member of your family diagnosed with diabetes?

⬜ No

⬜ Yes

If YES what is this person’s relationship to your child?

- …….. Father
- …….. Mother
- …….. Uncle / Aunt
- ……. Grandmother
- ……. Grandfather
- …….. Other ___________________

**2 pEDAGOGY**

**(a) Teacher Questionnaire**

1. **Gender** (please tick one)**:**

___ Male ___ Female

**2. Age Category** (please tick one)**:**

___ 25-29 ___ 30-34 ___ 35-39 ___ 40-49 ___ 50+

# 3. How would you rate your own level of physical activity? (please tick one)

___ Inactive - Less than 1 hour of activity/week eg gardening/leisurely walk

___ Moderately Active - Between 1-3 hours of moderate activity per week

___ Fairly Active - Between 3-5 hours of moderate activity /week

___ Quite Active - Between 1-5 hours of vigorous activity per week

___ Very active – Between 5-7 hours of vigorous activity per week

**4. How many times per week does your class do Physical Education lessons?** (please tick one)

___ 1-2 ___ 2-3 ___ 3-4 ___ 4-5 ___ 5 or more

**5. Please give an estimate of accumulative number of minutes on Physical Education lessons and Sport per week** (please write your answer below)**:**

Number of minutes = ______________

**6. Do you enjoy teaching Physical Education lessons?** (please tick one)**:**

___ Always ___ Mostly ___ Sometimes ___ Rarely ___ Never

**7. What are some of the issues that make teaching Physical Education in the primary school difficult?** (Please give an answer for each suggested reason by circling one of the numbers on sliding scale)

| Reason | Not important Very Important |
| --- | --- |
| Don’t like it | 1 2 3 4 5 |
| Behaviour management issues with this class | 1 2 3 4 5 |
| Lack of resources: space/equipment | 1 2 3 4 5 |
| Lack of expertise | 1 2 3 4 5 |
| Low priority | 1 2 3 4 5 |
| Crowded curriculum | 1 2 3 4 5 |
| Other? | 1 2 3 4 5 |

**8. Please rank the Key Learning Areas in terms of time given to planning and preparation** (rank from 1–8; 1 being the most time given, 8 being the least time given)**:**

KLA Ranking

Literacy _______

Numeracy _______

Science _______

Design and Technology _______

Physical Education and Health _______

SOSE _______

LOTE _______

The Arts _______

**9. What methods of assessment do you use, to monitor student progress and evaluate your Physical Education program?** (please tick one)**:**

­___ Observation

___ Physical tests

___ Checklists

___ None

___ Others (please specify):_________________________________________________

**10. For your class this term which Fundamental Motor Skills are you focusing on?** (please write your answer below)

___

___

**11. Please give an estimation of what percentage (%) of your program the following categories of activities would take up** (totalling 100%, please write your answer below)**:**

_____% - Fitness activities including running, skipping circuits

_____% - Gross Motor activities or Fundamental Motor Skills

_____% - Spatial awareness, Body awareness, Motor memory activities

_____% - Games – including cooperative games or games associated with sports.

# 12. Do you think it is important for you to lead a physically active life?

___ Yes ___ Unsure ___ No

**pEDAGOGY**

**(b) Teacher Observation DATA FORM**

**Verbal Instruction to whole class**

Keep a running watch on the duration of teacher instruction to the whole class through the lesson. Total time of instruction = ________mins

**Demonstration time**

How many times does the teacher demonstrate the activity to be learned by the students? For each demonstration of the skill by the teacher mark the boxes ⮽:

|  |  |  |  |  |  |  |  |  |  |  |  |  |  |  |  |  |  |  |  |
| --- | --- | --- | --- | --- | --- | --- | --- | --- | --- | --- | --- | --- | --- | --- | --- | --- | --- | --- | --- |
|  |  |  |  |  |  |  |  |  |  |  |  |  |  |  |  |  |  |  |  |

**Quality of Demonstration**

*Please indicate the quality of the teacher’s physical demonstration (please tick one):*

___ *Very poor quality or no demonstration*

*___ Reasonable quality demonstration at the beginning of the class*

*___ Good quality demonstration on more than one occasion in the class*

*___ Excellent quality demonstration through the class*

# Teacher - whole class communication tally

Please mark the boxes below according to the following instructions:

/ - Each time the teacher gives coaching cues or coach specific instruction to the class
X - Each time the teacher gives organisational or management instruction to the class

O - Each time the teacher speaks the class in general conversation / neither of the above

Each box represents approximately one episode of a minute in the class time:

|  |  |  |  |  |  |  |  |  |  |  |  |  |  |  |  |  |  |  |  |
| --- | --- | --- | --- | --- | --- | --- | --- | --- | --- | --- | --- | --- | --- | --- | --- | --- | --- | --- | --- |
|  |  |  |  |  |  |  |  |  |  |  |  |  |  |  |  |  |  |  |  |
|  |  |  |  |  |  |  |  |  |  |  |  |  |  |  |  |  |  |  |  |

**PEDAGOGY**

**(C )** TE**acher and individual student communication**

Each box represents an episode of approximately one-minute of teacher communication with the individual or small group of students, Mark boxes 🞣 or **−** for each time the teacher addresses an individual or small group of students. Marking 🞣 indicates positive communication with students while **−** indicates negative communication with students.

Teacher student communication spent teaching/coaching:

|  |  |  |  |  |  |  |  |  |  |  |  |  |  |  |  |  |  |  |  |
| --- | --- | --- | --- | --- | --- | --- | --- | --- | --- | --- | --- | --- | --- | --- | --- | --- | --- | --- | --- |
|  |  |  |  |  |  |  |  |  |  |  |  |  |  |  |  |  |  |  |  |

Teacher student communication spent managing:

|  |  |  |  |  |  |  |  |  |  |  |  |  |  |  |  |  |  |  |  |
| --- | --- | --- | --- | --- | --- | --- | --- | --- | --- | --- | --- | --- | --- | --- | --- | --- | --- | --- | --- |
|  |  |  |  |  |  |  |  |  |  |  |  |  |  |  |  |  |  |  |  |

## Total time of students communication spent teaching/coaching: ______mins

## Total time of students communication spent managing: ______mins

**3. EXAMPLE OF NUTRITION FORM FILLED IN BY NUTRITIONIST DURING INTERVIEW (HARD COPY ONLY ATTACHED)**
